# Gene expression patterns induced at different stages of rhinovirus infection in human alveolar epithelial cells

**DOI:** 10.1371/journal.pone.0176947

**Published:** 2017-05-30

**Authors:** Mohammad Reza Etemadi, King-Hwa Ling, Shahidee Zainal Abidin, Hui-Yee Chee, Zamberi Sekawi

**Affiliations:** 1 Department of Medical Microbiology and Parasitology, Faculty of Medicine and Health Sciences, University Putra Malaysia, UPM Serdang, Selangor DE, Malaysia; 2 Department of Biomedical Sciences, Faculty of Medicine and Health Sciences, Universiti Putra Malaysia, UPM Serdang, Selangor DE, Serdang, Selangor, Malaysia; 3 Genetics and Regenerative Medicine Research Centre (GRMRC), Faculty of Medicine and Health Sciences, Universiti Putra Malaysia, UPM Serdang, Selangor DE, Serdang, Selangor, Malaysia; University of Malaya, MALAYSIA

## Abstract

Human rhinovirus (HRV) is the common virus that causes acute respiratory infection (ARI) and is frequently associated with lower respiratory tract infections (LRTIs). We aimed to investigate whether HRV infection induces a specific gene expression pattern in airway epithelial cells. Alveolar epithelial cell monolayers were infected with HRV species B (HRV-B). RNA was extracted from both supernatants and infected monolayer cells at 6, 12, 24 and 48 hours post infection (hpi) and transcriptional profile was analyzed using Affymetrix GeneChip and the results were subsequently validated using quantitative Real-time PCR method. HRV-B infects alveolar epithelial cells which supports implication of the virus with LRTIs. In total 991 genes were found differentially expressed during the course of infection. Of these, 459 genes were up-regulated whereas 532 genes were down-regulated. Differential gene expression at 6 hpi (187 genes up-regulated vs. 156 down-regulated) were significantly represented by gene ontologies related to the chemokines and inflammatory molecules indicating characteristic of viral infection. The 75 up-regulated genes surpassed the down-regulated genes (35) at 12 hpi and their enriched ontologies fell into discrete functional entities such as regulation of apoptosis, anti-apoptosis, and wound healing. At later time points of 24 and 48 hpi, predominated down-regulated genes were enriched for extracellular matrix proteins and airway remodeling events. Our data provides a comprehensive image of host response to HRV infection. The study suggests the underlying molecular regulatory networks genes which might be involved in pathogenicity of the HRV-B and potential targets for further validations and development of effective treatment.

## Introduction

Human rhinovirus (HRV), a non-segmented positive sense RNA from *Picornaviridae*, [1], is the most prevalent human respiratory pathogen [2], consisted of three species, HRV-A, B and C [3]. HRV is associated with common colds and concomitant sinusitis [4] and otitis media episodes [5] as well as lower respiratory tract infection (LRTI) [6]. Bronchiolitis and pneumonia among young children, exacerbations of asthma in older children, and exacerbations of chronic obstructive pulmonary disease (COPD) and pneumonia in older age are the predominant features associated with HRV at different age groups [7,8]. Association of HRV with LRTIs has also been demonstrated in lower airway samples [9,10]. Human airway epithelial cell is the primary target for HRV infection leading to interstitial pneumonia [11,12]. An emerging body of evidence indicates that asthma exacerbations and COPD triggered by HRV alterations of pulmonary epithelial cells and subsequent airway inflammation rather than direct cytotoxicity on the cells [13,14].

Global transcriptional response of HRV-infected pulmonary epithelial cells has been investigated using *in vitro* [15,16] and *in vivo* [17] studies, however, the focus of the studies were mainly on a single species of HRV-A. Although clinico-epidemiological studies have revealed specific features associated with HRV species [18,19], the transcriptomic data is rather limited and have not been extensively understood with respect to different species [20]. In the current study we hypothesized that HRV-B infection could deregulate expression of genes in a timely manner with potential to introduce novel targets attributed to species B for further investigations. To address this hypothesis, we performed gene expression analysis of the RNA samples obtained from alveolar epithelial cells infected with of HRV-B using Affymetrix Genechip technology. Our study reveals comprehensive gene expression profile of epithelial cell response to HRV-B infection across different time points *in vitro*. We have demonstrated remarkable gene expression changes in each time points post infection including novel virus-induced genes. Collectively, our data provides novel targets for further evaluation and to develop efficient anti-HRV therapeutic strategies.

## Materials and methods

### Preparation of virus stock

Serotype 72 of HRV-B species was purchased from ATCC (ATCC VR-1182). H1HeLa cell line (ATCC CRL-1958) was grown in Leibovitz’s L-15 medium supplemented with 10% (v/v) fetal bovine serum (FBS), penicillin (IU/ml), streptomycin (100 μg/ml) and amphotericin B (2 μg/ml) and incubated at 37°C under CO_2_ free incubator. Stocks of HRV72 were propagated in H1Hela cells. H1Hela monolayer cells at 80% confluency were inoculated with HRV72 and virus absorption was carried out for 1.5 hours with gentle shaking. The cells were nourished with fresh media with 2% FBS and incubated at 34°C. The cell culture flasks were monitored daily and when HRV typical CPE covered more than 50% of the cells, they were harvested after three freeze-thaw cycles to rupture the membranes followed by centrifugation to pellet cell debris and stored at -80°C. Then the crude virus was concentrated using ultracentrifugation at 48k rpm for 2 hours using OptiSeal Polypropylene Tubes (optima L-100XP ultracentrifuge; Beckman Coulter, Inc.). The suspended impure virus was subjected to sucrose gradient using Beckman SW41 rotor to remove exogenous proteins and contaminants. The sucrose fractions were collected by peristaltic pump and analyzed using SDS-PAGE. SDS-PAGE analysis of sucrose fractions revealed that pure virus was present in high concentration at UP6 fraction as evident by the presence of three distinct bands corresponding to VP1-3 capsid proteins (S1 Fig). The fractions containing concentrated intact virus were dialyzed using 3500 MWCO Slide-A-Lyzer Dialysis cassettes G2 (3.5 ml) and subsequently followed by concentration step using concentrator 9K MWCO (Thermo Fisher Scientific Inc.) and aliquots were stored at -80°C.

### Infectious center assay

H1Hela cells were plated over 6-well plates at a cell density of 4–5 × 10^5^ cells per well and incubated overnight at 37°C. Tenfold virus suspensions were inoculated in duplicates and absorbed for 1.5 hrs. Then the inoculum was replaced with agarose overlay containing 1.5% of carboxymethylcellulose sodium salt (low viscosity; C5678 Sigma-Aldrich), magnesium chloride (0.04 M) and 2% FBS. When the layer was solidified the plates were turned upside-down and incubated for 5 days at 34°C. The cells were fixed with 3.7% formaldehyde and stained with 0.1% crystal violet solution. The number of plaques at each dilution was calculated as PFU [21,22]. The titer of the purified virus was 5.2 × 10^9^ PFU/mL, as depicted in S2 Fig.

### Cell viability

A human adenocarcinomic alveolar basal epithelial cell line, A549, (ATCCCCL-185) was cultured in F12K medium (ATCC). A549 cells were seeded in 96-well plates at density of 12,500 cells/cm2 and incubated at 37°C in humidified incubator with 5% CO2 for overnight and then infected with various MOI (1, 5, 10, 15, 25, 30, 35 and 40) and incubated at 34°C. Cell viability was assessed at 24 and 48 hpi by adding 3-(4, 5-dimethylthiazol-2-yl)-2, 5-diphenyltetrazolium bromide (MTT) solution to each well and incubated for 4 hours in the dark condition. Then stopping solution was added to each well and incubated for 20 minutes. The absorbance was measured at 570 nm using a micro plate reader. Cell viability was also tested by Trypan blue exclusion method [23]. A549 cells where seeded in 25 cm^2^ culture flasks and infected with virus MOI of 10 and 50. At 24 and 48 hpi the supernatant was centrifuged and the cells were trypsinized and collected from both infected and mock-infected flasks. The viable cells where counted under the phase contrast microscope using 0.4% (w/v) Trypan blue dye aqueous solution.

### Infection of epithelial cells and total RNA preparation

The A549 cells grown in 25 cm^2^ culture flasks were infected with HRV72 at multiplicity of infection (MOI) of 10 or mock-infected at three independent replicates with a week interval [24]. Total mRNA was extracted from both HRV72-infected and mock-infected A549 cells using GeneJET RNA purification kit (Thermo Fisher Scientific Inc., Germany), according to the manufacturer’s instructions. DNA contamination was removed using DNase I treatment (Worthington Biochemical Corporation). RNA concentration (at 260 nm) and purity (260/280 nm and 260/230 nm ratio) was measured by using a Thermo Scientific NanoDrop 1000 Spectrophotometer. The RNA integrity and DNA removal was confirmed by 1% denaturating agarose gel electrophoresis. The proportion of the full-length RNA was evaluated by microfluidic analysis using Agilent 2100 Bioanalysier using RNA LabChip Kit (Germany), as per manufacturer’s instructions. The samples with RNA Integrity Number (RIN) of 7 and greater were used for microarray hybridization.

### Virus quantification

Viral RNA was extracted from cell culture supernatants at 6, 12, 24, and 48 hpi and also from third PBS wash of the cell monolayer before collecting the cells using GeneJET Viral DNA and RNA Purification Kit. Equal amounts of the purified RNA from both supernatant and cell lysate samples were reverse transcribed to obtain cDNA using Maxima First Strand cDNA Synthesis Kit (Thermo Fisher Scientific Inc., Germany). Universal Probe Library Assay design centre (https://lifescience.roche.com) was used to design primers to target a 94 nt region at IRES sequence (Table 1) to determine viral load in both samples using LightCycler480 Instrument II (Roche Life Science). Standard curve was generated by 5-fold dilutions of cDNA template of virus positive control and used to determine relative quantities of virus by extrapolating the Cp values of unknown samples against the linear regression. Expression of the target virus was normalized to the expression of the *PSMB2* as housekeeping gene.

**Table 1:** Reference position and primer set used for microarray data validation using real-time PCR.

Functional group	Gene name	Amplicon size	Primers (5'→3')	UPL Probe no
chemokine	CXCL8	90	F: atggttccttccggtggt	72
			R: gagcactccataaggcacaaa	
	CCL20	90	F: atgtgctgtaccaagagtttgc	71
			R: gcagtcaaagttgcttgctg	
Anti-apoptotic	BCL2A1	64	F: accagcataggtgtgtgattgt	75
			R:caggagaatggataaggcaaa	
Transcription factor	FOSL1	94	F: ctccggttcctgcacttg	69
			R: ggccttgtgaacagatcagc	
	JUN	62	F: ctgtccctctccactgcaac	19
			R: ccaaaggatagtgcgatgttt	
	EGR1	92	F: ggtttggctggggtaactg	22
			R: agccctacgagcacctgac	
Signal transduction	DUSP6	112	F: ttggaacttactgaagccacct	66
			R: cgactggaacgagaatacgg	
	GNB4	106	F: tctgcacgaaggtcaaagag	21
			R: cgggacatgtctcagatatcaa	
Interferon type I	IFNB1	67	F: cgacactgttcgtgttgtca	25
			R: gaagcacaacaggagagcaa	
	IFNB1[16]	83	F: aggacgccgcattgacc	
			R: attccagccagtgctagatga	
Interferon type III	IL29[16]	83	F: tggtgctggtgactttggtg	
			R: gcagcccttcccagttgt	
	IL28[16]	93	F: gctgaccgtgactggagca	
			R: gacagggacttgaactgggcta	
Housekeeping	GAPDH	66	F: gcccaatacgaccaaatcc	60
			R: agccacatcgctcagacac	
HRV72	IRES	94	R: Gacccagacaccactggatt	60
			F: gactcgcatgtgcttgattg	

Abbreviations: bp: base pairs

### Microarray hybridization

Three independent replicates of RNA samples from mock-infected and HRV-infected A549-cells at MOI of 10 PFU were purified at 6, 12, 24, and 48 hpi and hybridized to microarray genechips for gene expression study. The samples were performed in Affymetrix GeneChipPrimeView Human Genome U133 Plus 2.0 microarray system includes more than 53,000 probes covering more than 20,000 genes and processed according to the manufacturer’s instructions (Affymetrix, Inc, USA). To achieve reproducible results, 100 ng of input RNA with RNA Integrity Number (RIN) of minimum 7.0 was applied as a standard amount for first-strand cDNA synthesis (at 42°C for 2 hours) primed with a T7-oligo (dT) primer using GeneChip 3’ IVT Express kit (P/N 901228, Affymetrix, USA). Second-strand cDNA was subsequently synthesized using DNA polymerase and RNase H (at 16°C for 1 hour). Linear RNA amplification using T7 transcription technology was employed in this kit. In the next step, the double-stranded cDNA was used as a template to amplify biotin-modified RNA (amplified RNA or aRNA). The aRNA was purified and concentration and purity was measured by using NanoDrop Spectrophotometer. To achieve optimal sensitivity of the assay, the labelled aRNA was fragmented to a mean size of 200 bases at 94°C and for 35 minutes before hybridization on gene chip array. Hybridization, staining and scanning of GeneChips were performed in Research Instruments Sdn. Bhd., Malaysia, using GenChip Hybridization, Wash, and Stain Kit (P/N 900720), according to the manufacturer’s instructions. Hybridization cocktail composed of 10 μg of fragmented aRNA and hybridization controls was hybridized to the GeneChip at 45°C for 16 hours and 60 rpm rotation in hybridization oven. Then the arrays were washed and stained in GeneChip Fluidics Station 450 prior to scanning with GeneChip Scanner 3000 (Affymetrix, Santa Clara, CA, USA).

### Microarray data processing

The array fluorescent signals were obtained as DAT files and converted to probe intensity data (.CEL) files using Gene Chip Command Console (AGCC) Software (instrument control software available at: https://www.thermofisher.com/us/en/home/life-science/microarray-analysis/microarray-analysis-instruments-software-services/microarray-analysis-software.html). CEL files were normalized to remove effects related to systematic variations. Summarized expression values (CHP files) were created using Robust Multichip Analysis (RMA) summarization algorithm [25] implemented in Affymetrix Expression Console Software 1.3 (available at: https://www.thermofisher.com/us/en/home/life-science/microarray-analysis/microarray-analysis-instruments-software-services/microarray-analysis-software.html). RMA workflow is based on quantile normalization of probe level signal intensities and has a general background correction to reduce interarray variability. The constructed CHP files were used for evaluation of individual hybridization success (QC control) and identification of outlier sample using Principle Component Analysis (PCA) in Affymetrix Expression Console Software 1.3. The quality of the starting RNA samples were evaluated using internal control housekeeping genes (GAPDH and ACTB). Entire target labelling process was monitored by adding four exogenous, premixed control spikes: lys, phe, thr, and dap. Efficiency of sample hybridization on the array was assessed by hybridization controls (bio B, bio C, bio D, and cre). QC analysis using Expression Console software showed that all the samples were located within threshold boundaries. The RNA samples hybridized to the array had RNA Integrity Number (RIN) of 7 or greater. The 3′/5′ ratios of internal control probe sets including GAPDH and ACTB were less than 3 for all the arrays indicating appropriate sample quality [26]. The signal value profile was as expected with correct relative abundance for each of the spikes for both target labelling and sample hybridization indicating acceptable hybridization process. Normalized and log_2_ transformed data were used to identify differentially expressed genes (DEG) using one-way analysis of variance (ANOVA) and to construct heat-map dendrogram and hierarchical clustering in virus-infected, as compared to the mock-infected cells using Transcriptome Analysis Console v2.0 (available at: https://www.thermofisher.com/us/en/home/life-science/microarray-analysis/microarray-analysis-instruments-software-services/microarray-analysis-software.html). The data was filtered to select DEGs using a p-value of 0.05 and less with a ≥1.5-fold or ≤-1.5-fold cut-off. The microarray data have been submitted to Gene Expression Omnibus (GEO, https://www.ncbi.nlm.nih.gov/geo/) and assigned the accession number GSE87463.

### Functional analysis

Functional Annotation Cluster tool provided by Database for Annotation, Visualization and Integrated Discovery (DAVID) bioinformatics resources 6.7, at NIAID/NIH, (available at: http://david.abcc.ncifcrf.gov/) was used for gene annotation and to identify functional categories enriched in differentially expressed genes at each time points. Modified Fisher’s exact test (EASE score of ≤ 0.01) was used to determine the probability of overexpressed gene ontology (GO) biological process terms. The tables were arranged according to enrichment score of ≥ 1.5.

### Validation of microarray results by qRT-PCR

Representative differentially expressed genes from different functional groups including chemokine (CXCL8, CCL20, CXCL3), anti-apoptotic (BCL2A1), transcription factors (FOSL1, JUN, and EGR1) and signal transduction mediators (DUSP6 and GNB4) were selected to further validate by qRT-PCR. The expression profiles of the IFN genes including IFN-β, Il29 (IFN-λ1), and IL28 (IFN-λ2/λ3) were further evaluated using qRT-PCR. Universal Probe Library (Roche: https://lifescience.roche.com/) was used to design primers and probes (Table 1). In order to estimate PCR efficiency, standard curves were derived from 5×serial dilutions for all genes. The slop of standard curves was used to calculate PCR efficiency. The quantification was performed using LightCycler 480 Instrument II and LightCycler LC480 software version LCS480 1.5.0.39 under following settings: 95°C for 10 min denaturation followed by 45 cycles of 95°C for 10 sec, annealing at 60°C for 30 sec, 72°C for 30 sec. Comparative results were calculated and the expression of genes were normalized to the GAPDH in all samples at 6, 12, 24, and 48 hpi. The amplified products were further analyzed by agarose gel and also subjected to the melting curve analysis (65 to 95°C) at the end of the experiment to determine the specificity of the primers.

### Statistical analysis

Data were analyzed using the Statistical Package for the Social Sciences (SPSS) version 18.0. All *P*-values were two-tailed and values of < 0.05 were considered as statistically significant. One-way analysis of variance (ANOVA) was applied to identify differentially expressed genes (DE genes). ANOVA with post hoc comparison using Tukey test was used to compare mean viral load between time points. Student’s independent sample t-test was applied to each data set for validation study by qRT-PCR. The data were expressed as the means ± SEM.

## Results

### High viability of airway epithelial cells was noticed across the HRV infection

**Fig 1:**
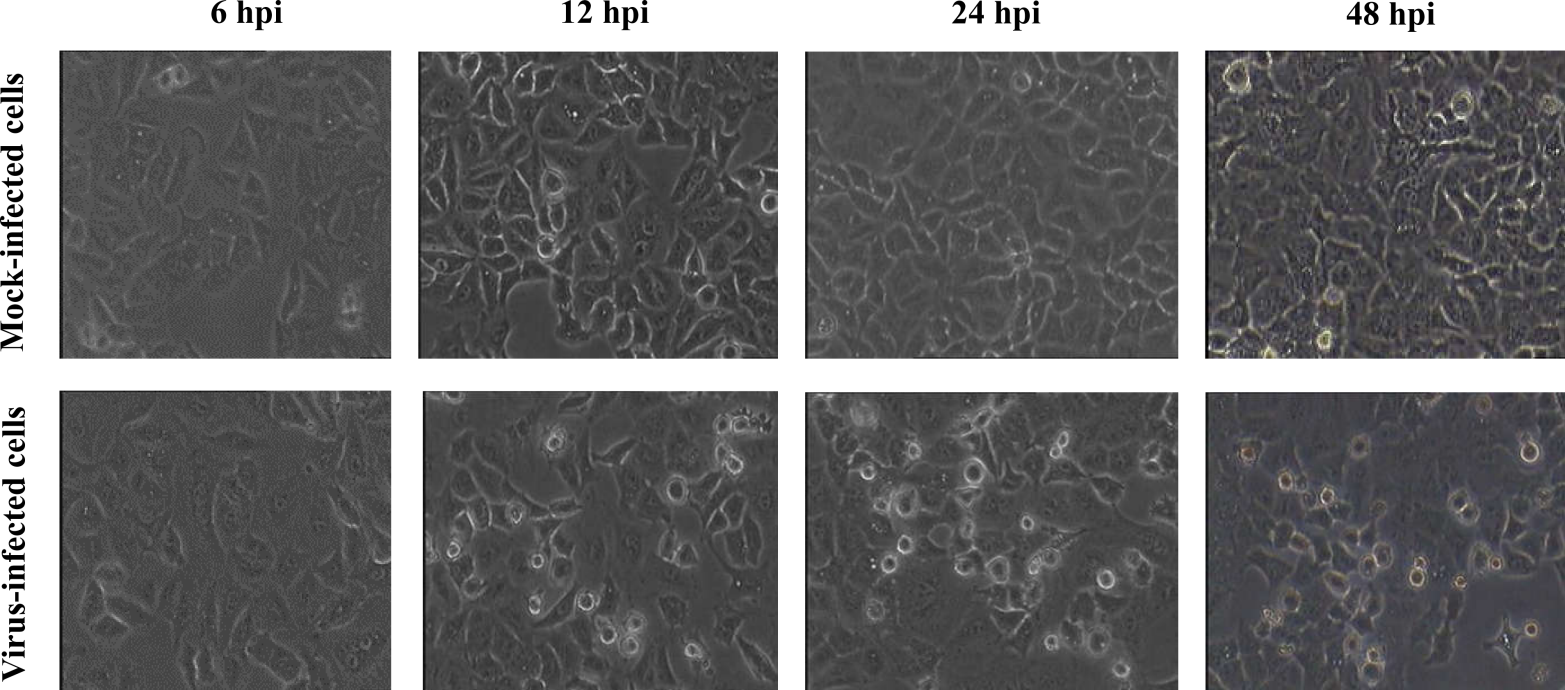
Infection of A549 cells with HRV72. Representative microphotographs of A549 cells either mock-infected (upper) or infected with HRV72 (down). HRV72 was inoculated at MOI of 10 onto A549 cells for 1.5 hrs. Then cells were incubated at 34°C and monitored for CPE development up to 48 hpi. The cells were visualized using a phase contrast microscope at 200× magnification.

Clear and countable virus plaques were optimized in the presence of magnesium chloride. To comprehend whether HRV72 infection induces any cytotoxic effects, the viability of A549 cells was determined using MTT assay. The viability decreased as MOI increased and at MOI of 10 the cell viability was 95% at 6 and 12 hpi, and subsequently decreased to 86% and 69% at 24 and 48 hpi, respectively (S3 Fig). Trypan blue dye exclusion test also demonstrated high viability of the virus infected cells with 97% and 91% viability at 24 and 48 hpi, respectively. The A549 cell monolayers were inoculated with HRV72 at MOI of 10 PFU per cell, which was suggested as a peak viral titer in nasal samples of patients with HRV cold in another study [15]. The time-course analysis of HRV72 infection in A549 cells was performed by applying this infection protocol. The virus induced specific cytopathic effect (CPE) characterized by shrinkage and rounding off the cells as well as detachment and destruction of cell layers, as observed by light microscopy (Fig 1). CPE started to be appeared at 12 hpi on A549 cells and continued to increase up to 48 hpi.

### HRV increased in a time-dependent manner across the infection course

**Fig 2:**
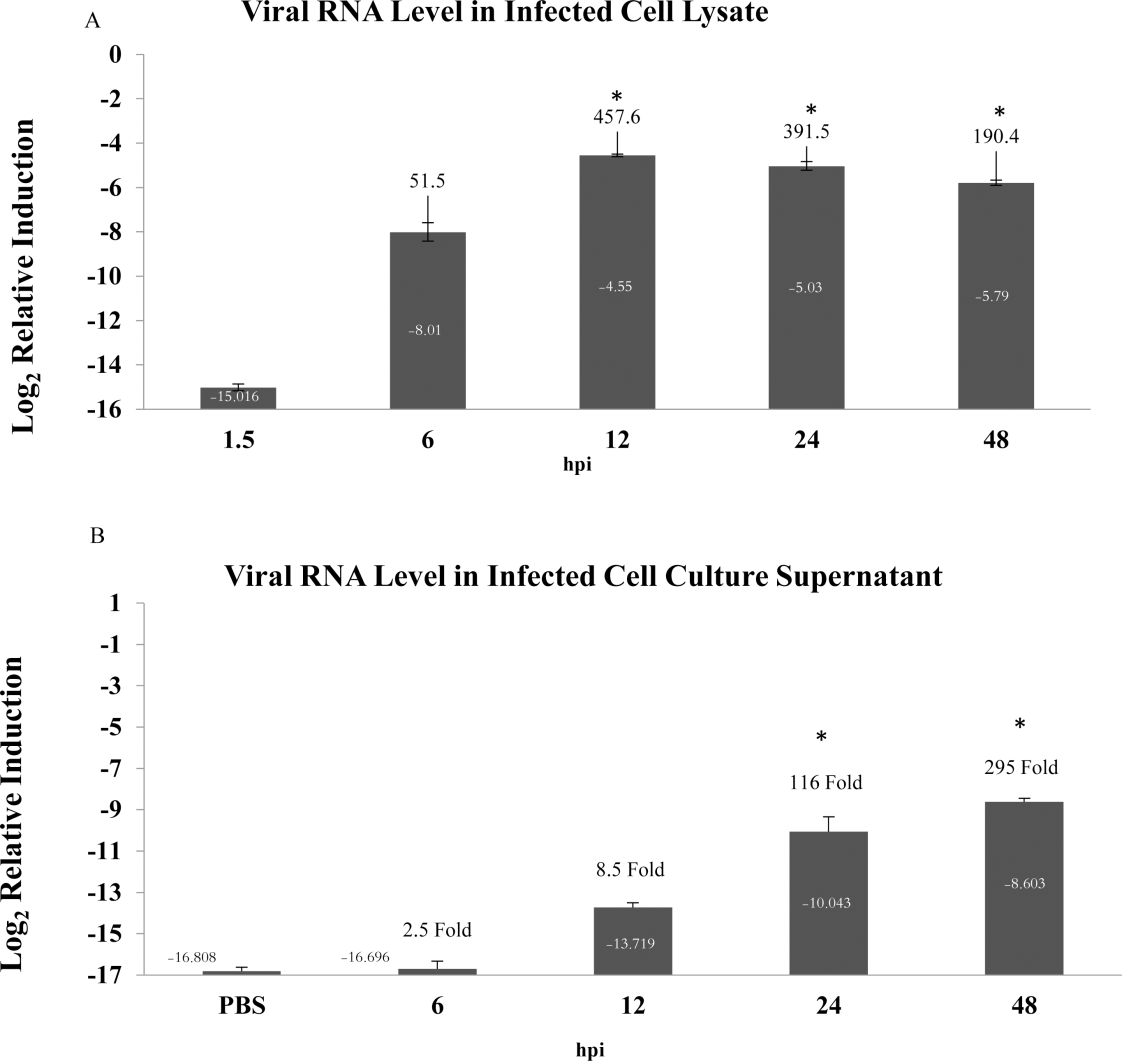
Time-dependent analysis of HRV72 propagation. **(A)** Determination of viral RNA level changes in HRV72 infected A549 cells. **(B)** Determination of viral RNA level changes in supernatant of HRV72 infected A549 cells. QRT-PCR was performed using primers specific for both HRV72 and Psmb2 at indicated time points and sample types. The amount of the HRV72 RNA was normalized to the amount of Psm2 mRNA. The relative abundance was calculated by standard curve method. Values on the y-axis are presented after log_2_ transformation. The induction of viral RNA relative to mock-infected cells is shown on a log_2_ scale. Each error bar represents standard deviation of mean viral RNA level from three independent experiments. Fold changes are indicated for each time compared with 1.5 hpi in Fig A and PBS in Fig B. *: a significant increase compared with 1.5 hpi (p<0.05, by post hoc comparison using Tukey test); hpi: hours post infection.

In order to confirm the active replication of the virus in A549 cells, the level of viral RNA was determined in samples collected from both cell lysate and cell culture supernatant at indicated time points. The cell lysate of the 1.5 hour post-infected monolayer cells was used as the baseline in virus quantification analysis. The third PBS wash was used as a baseline for virus quantification in supernatant. As shown in Fig 2. A, a significant increase of viral RNA in HRV72-infected A549 cell lysate was noticed at 12, 24, and 48 hpi compared to baseline at 1.5 hours of inoculation (*p* ≤ 0.001). Multiple comparisons also indicated significant differences among all time points (6, 12, 24, and 48 hpi) in any combination (*p* ≤ 0.001). The substantial fold change was started at 6 hpi (51.5 fold); continued at 48 hpi (190.4 fold) and highest viral load was observed at 12 hpi (457.6 fold). Analysis of the virus propagation in cell culture supernatant also demonstrated time-dependent increase of viral RNA up to 48 hpi (Fig 2B). Significant induction of viral RNA was observed at 24 and 48 hpi (*p* ≤ 0.001) with highest RNA fold at 48 hpi (295 Fold). According to the data acquired from experiments analyzing both intracellular and extracellular viral RNA level, it is apparent that HRV72 achieved higher viral loads during early time points after infection (highest at 12 hpi). This is in line with shorter incubation period in HRV infections compared to the other respiratory viruses [19].

### HRV infection modified large number of genes at timely manner

**Fig 3:**
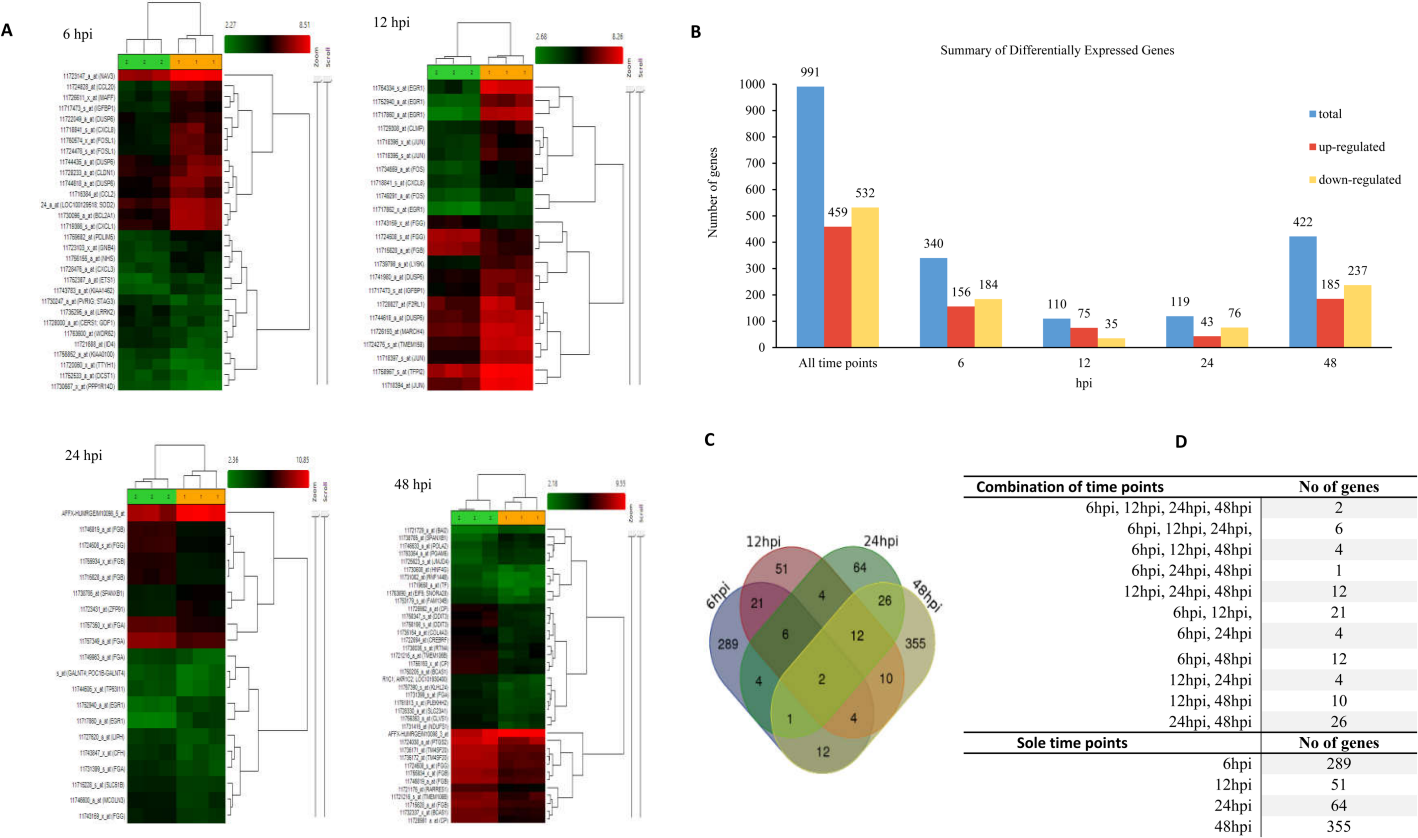
Gene expression analysis of the A549 cells infected with HRV. Total RNA was extracted from both HRV-infected and mock-infected A549 cells and hybridized to GeneChip PrimeView GeneChip Arrays. (A) Hierarchical cluster and heat map analysis of HRV-affected genes. Horizontal clusters shows genes and clusters in the columns represent samples (samples represent by 1 and orange color are virus infected and samples show by 2 and in green represent mock-infected samples). The DEGs in response to HRV infection were filtered here at p-value ≤ 0.05 and with a fold change of two and above (One-Way Between-Subject ANOVA (unpaired)). Genes shown in red were up-regulated, while genes shown in green were down-regulated, in HRV-infected compared to mock-infected cells. (B) Summary of DE genes induced by HRV72. The DE genes were determined at four time points by filtering of the genes at *p-*value ≤ 0.05 and with a fold change of 1.5. (C) Venn diagram showing overlap of DEGs in HRV-infected cells compared with mock-infected A459 cells with 1.5-fold (p-value ≤ 0.05). (D) Number of overlap genes in different combination of time points are presented in the table.

The affymetrix microarray gene expression analysis was performed at four time points post infection because the time course analysis of the infection demonstrated that replication of this virus is time-dependent (Fig 2). A comparative analysis of three independent experiments between HRV-infected and mock-infected cells using a threshold of 1.5-fold identified collectively 991 differentially expressed (DE) genes across all time points, from which 459 (46%) were up-regulated and 532 (54%) were down-regulated. During infection down-regulated genes were surpassed up-regulated genes and the category of up-regulated genes consisted of largest fold changes. The samples were separated in two groups including virus-infected and mock-infected based on hierarchical clustering of gene expression patterns, as shown in Fig 3. A. Surprisingly, many genes were significantly affected at 6 hpi and continued to a less extent at 12 and 24 hpi but again increased substantially at 48 hpi (Fig 3B). Complete list of DE genes are provided in S1–S4 Tables. Using a more stringent 2-fold criterion the up- and down-regulated DE genes across four time pointes were combined and presented as separate gene list in Tables 2 and 3, respectively.

**Table 2:** Selected genes markedly up-regulated by HRV infection at different time points.

No	Gene symbol	Description	Fold change^b^	*p-*value^a^
Up-regulated genes at 6 hpi				
1	CCL20	Chemokine (C-C motif) ligand 20	5.41	0.0010
2	FOSL1	FOS-like antigen 1	3.26	0.0025
3	BCL2A1	BCL2-related protein A1	3.06	0.0002
4	CXCL8	Chemokine (C-X-C motif) ligand 8	3.04	0.0097
5	MAFF	V-maf avian musculoaponeurotic fibrosarcoma oncogene homolog F	2.98	0.0027
6	CXCL1	Chemokine (C-X-C motif) ligand 1	2.83	0.0006
7	DUSP6	Dual specificity phosphatase 6	2.8	0.0135
8	IGFBP1	Insulin-like growth factor binding protein 1	2.58	0.0011
9	NHS	Nance-Horan syndrome (congenital cataracts and dental anomalies)	2.53	0.0063
10	CLDN1	Claudin 1	2.45	0.0083
11	PDLIM5	PDZ and LIM domain 5	2.3	0.0009
12	LOC100129518	Uncharacterized LOC100129518; superoxide dismutase 2, mitochondrial	2.23	0.0014
13	NAV3	Neuron navigator 3	2.21	0.0006
14	CCL2	chemokine (C-C motif) ligand 2	2.2	0.0154
15	ETS1	V-ets avian erythroblastosis virus E26 oncogene homolog 1	2.1	0.0492
16	CXCL3	Chemokine (C-X-C motif) ligand 3	2.05	0.0150
17	KIAA1462	KIAA1462	2.04	0.0066
18	GNB4	guanine nucleotide binding protein (G protein), beta polypeptide 4	2.02	0.0133
Up-regulated genes at 12 hpi				
1	EGR1	Early growth response 1	24.96	0.0000
2	JUN	Jun proto-oncogene	3.26	0.0000
3	TMEM158	Transmembrane protein 158 (gene/pseudogene)	3.06	0.0000
4	FOS	FBJ murine osteosarcoma viral oncogene homolog	2.9	0.0004
5	CLMP	CXADR-like membrane protein	2.53	0.0023
6	F2RL1	Coagulation factor II (thrombin) receptor-like 1	2.47	0.0401
7	IGFBP1	Insulin-like growth factor binding protein 1	2.28	0.0009
8	MARCH4	Membrane-associated ring finger (C3HC4) 4, E3 ubiquitin protein ligase	2.23	0.0001
9	DUSP6	Dual specificity phosphatase 6	2.23	0.0002
10	LY6K	Lymphocyte antigen 6 complex, locus K	2.15	0.0003
11	TFPI2	Tissue factor pathway inhibitor 2	2.09	0.0018
12	CXCL8	Chemokine (C-X-C motif) ligand 8		
Up-regulated genes at 24 hpi				
1	EGR1	Early growth response 1	5.34	0.0019
2	ZFP91	ZFP91 zinc finger protein	3.26	0.0000
3	SPANXB1	SPANX family, member B1	2.08	0.0003
Up-regulated genes at 48 hpi				
1	SPANXB1	SPANX family, member B1	4.26	0.0028
2	POLA2	polymerase (DNA directed), alpha 2, accessory subunit	2.41	0.0125
3	BAI2	brain-specific angiogenesis inhibitor 2	2.4	0.0014
4	PGAM5	phosphoglycerate mutase family member 5	2.1	0.0466
5	JMJD4	jumonji domain containing 4	2.03	0.0160

DEGs were generated by Transcriptome Analysis Console v2.0.

^a^ *p*-values obtained from One-Way Between-Subject ANOVA (unpaired)

^b^ The highest fold change was selected for those genes had more than one probe set induced in similar expression pattern.

**Table 3:** Selected genes markedly down-regulated by HRV infection at different time points.

No	Gene symbol	Description	Fold change ^b^^,^ ^c^	p-value ^a^
Down-regulated genes at 6 hpi				
1	ID4	inhibitor of DNA binding 4, dominant negative helix-loop-helix protein	-2.02	0.012324
2	TTYH1	tweety family member 1	-2.02	0.016162
3	PPP1R14D	protein phosphatase 1, regulatory (inhibitor) subunit 14D	-2.06	0.003686
4	KIAA0100	KIAA0100	-2.07	0.00537
5	WDR62	WD repeat domain 62	-2.08	0.021351
6	DCST1	DC-STAMP domain containing 1	-2.08	0.038792
7	CERS1	ceramide synthase 1; growth differentiation factor 1	-2.11	0.029393
9	PVRIG	poliovirus receptor related immunoglobulin domain containing; stromal antigen 3	-2.19	0.04029
10	LRRK2	leucine-rich repeat kinase 2	-2.21	0.017273
Down-regulated genes at 12 hpi				
1	FGB	Fibrinogen beta chain	-2.2	0.002607
2	FGG	Fibrinogen gamma chain	-2.4	0.000447
Down-regulated genes at 24 hpi				
1	SLC51B	Solute carrier family 51, beta subunit	-2.01	0.000156
2	GALNT4	Polypeptide N-acetylgalactosaminyltransferase 4; POC1B-GALNT4 readthrough	-2.03	0.028953
3	LIPH	Lipase, member H	-2.04	0.029327
4	TP53I11	Tumor protein p53 inducible protein 11	-2.08	0.01715
5	FGG	Fibrinogen gamma chain	-2.09	0.002158
6	CFH	Complement factor H	-2.15	0.00602
7	MCOLN3	Mucolipin 3	-2.29	0.014979
8	FGA	Fibrinogen alpha chain	-2.44	0.03468
9	FGB	Fibrinogen beta chain	-2.53	0.000162
Down-regulated genes at 48 hpi				
1	FGB	fibrinogen beta chain	-2.36	0.000151
2	DDIT3	DNA-damage-inducible transcript 3	-2.39	0.001531
3	BCAS1	breast carcinoma amplified sequence 1	-2.42	0.000624
4	DDIT3	DNA-damage-inducible transcript 3	-2.47	0.000572
5	KLHL24	kelch-like family member 24	-2.47	0.005459
6	TMEM106B	transmembrane protein 106B	-2.48	0.229047
7	EIF5	eukaryotic translation initiation factor 5; small nucleolar RNA, H/ACA box 28	-2.55	0.026405
8	TM4SF20	transmembrane 4 L six family member 20	-2.56	0.000003
9	CP	ceruloplasmin (ferroxidase)	-2.66	0.000003
11	FGA	fibrinogen alpha chai	-2.76	0.004108
12	RNF144B	ring finger protein 144B	-2.86	0.031129

DEGs were generated by Transcriptome Analysis Console v2.0.

^a^ p-values obtained from One-Way Between-Subject ANOVA (unpaired)

^b^ The lowest fold change was selected for those genes had more than one probe set induced in similar expression pattern.

^c^Negative numbers show down-regulation in HRV-infected cells compared with mock-infected cells

The overlaps of DE genes have been revealed across all-time points using Venn diagrams as shown in Fig 3C. During infection majority of the genes uniquely expressed in each time points (759 genes out of 991, 76.6%) and collectively less overlap of genes (23.4%) was found, which has been shown individually between time points as in Fig 3D (the complete list of overlap genes is presented in S5 Table). The minority of the affected genes at 6 hpi were common to other time points (50 out of 339 DE genes, 14.7%), with 41% (21 out of 51) in common with 12 hpi and 8% (4 out of 50) and 24% (12 out of 50) in overlap with 24 and 48 hpi, respectively and the rest were common in other combinations. Of 110 DE genes, 59 genes (53.6%) were overlapped including 35.6% (21 out of 59 DE genes) overlapped with 6hpi, and 7% (4 genes) and 17% (10) were common in 24 and 48 hpi, respectively. Of the 119 DE Genes, 64 genes (54%) uniquely expressed at this time points and the overlap genes are presented in Fig 3C and 3D. Taken together, the data revealed that HRV infection has significant effect on global gene expression and majority of DE genes were unique at each time point.

### Diverse functional groups were enriched by HRV during the infection course

**Fig 4:**
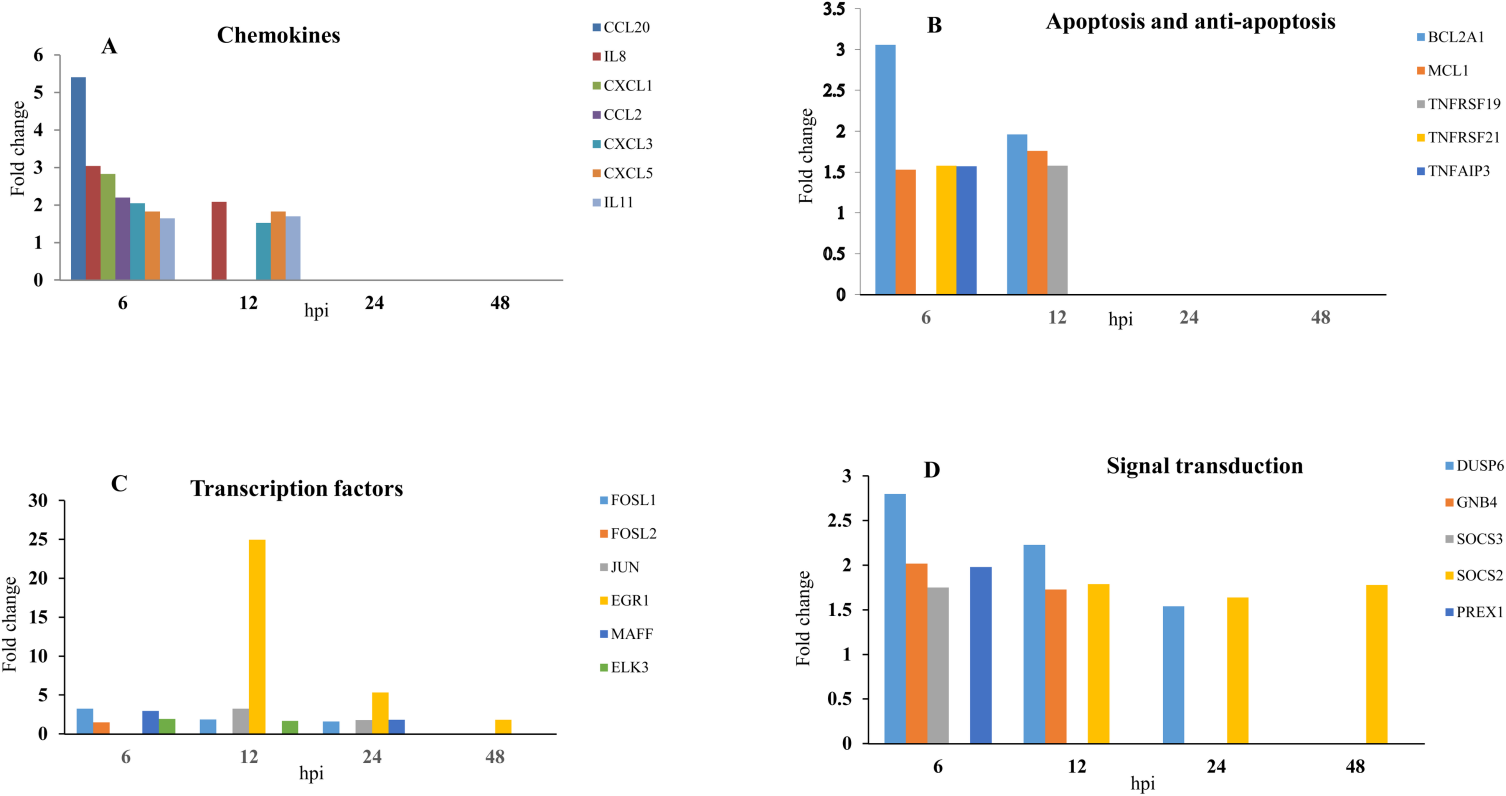
Changes in the expression of the candidate genes related to different functional groups at different time points. A) Fold expression of chemokines. B) Fold expression of apoptosis and anti-apoptosis genes. C) Fold expression of transcription factors. D) Fold expression of signal transduction factors. Fold change was presented as the ratio of gene expression level of HRV-B72-infected versus mock-infected cells.

The DE genes with known and available functional annotations where focused for functional analysis using analysis Gene Ontology classification (DAVID). Diverse functional categories were enriched across the infection course are presented at Table 4 (p-value ≤ 0.5 and an enrichment score ≥ 1.5). Analysis of the DE genes at 6 hpi revealed significant enrichment of functional clusters that were associated with inflammatory response and chemokine activity. HRV72 infection resulted in the sudden induction of many chemokines and proinflammatory genes both in numbers and magnitude. HRV72 induced a mixture of CC (CCL2, CCL20) and CXC (CXCL1, CXCL3, CXCL5, CXCL8) chemokines. All of them showed maximum fold change at 6 hpi. Expression of CXCL8, CXCL3, and CXCL5 continued up to 12hpi and the rest of chemokines exclusively expressed at 6hpi (Fig 4A).

**Table 4:** Functional classification of DEGs by HRV infection of A549 cells at different time points.

Functional group term ^a^	Enrichment Score	No of genes ^b^	% ^c^	*p-*value ^d^	Genes
Functional terms at 6 hpi					
Chemotaxis	2.48	9	7.89	0.006004559	CXCL1, CCL2, IL8, IL16, CXCL5, CCL20, CXCL3, FOSL1, PLAUR
Inflammatory response	2.48	5	7.81	0.00876546	CXCL1, CCL2, IL8, CCL20, CXCL3
Chemokine signaling pathway	2.48	8	7.02	0.016583169	CXCL1, CCL2, IL8, CXCL5, CCL20, CXCL3, PREX1, GNB4
Functional terms at 12 hpi					
Wound healing	3.7166	13	13.3	4.40E-07	DCBLD2, F2RL1, ELK3, PLAUR, IL11, FGG, THBD, FGA, EREG, FGB, HMOX1, IGFBP1, TFPI2
Regulation of apoptosis	2.937	15	15.3	0.015904881	CADM1, SOCS2, MCL1, BCL2A1, FOXO1, SART1, CDKN1B, SFRP1, NUPR1, HMOX1, JUN, ALDH1A3, TGM2, TNFRSF19, FOSL1
Anti-apoptosis	2.937	7	7.1	0.045276625	SOCS2, SFRP1, MCL1, HMOX1, BCL2A1, TGM2, FOXO1
Vasculature development	2.416	8	8.2		IL8, EREG, HMOX1, JUN, TGM2, FOXO1, ELK3, CYR61
Hemostasis	2.14	8	8.2	4.77E-04	FGG, THBD, FGA, FGB, F2RL1, TFPI2, IL11, PLAUR
Complement and coagulation cascades	2.14	6	6.1	0.009740322	FGG, THBD, FGA, FGB, CFH, PLAUR
Functional terms at 24 hpi					
Extracellular space	2.73	11	18.3	0.004	ZFP91, FGG, FGA, EREG, FGB, CFH, APOH, CD70, FGL1, CP, BMP6
Fibrinogen complex	2.327	4	6.7	1.81E-04	FGG, FGA, FGB, FGL1
Functional terms at 48 hpi					
Extracellular space	4.57	13	10.8	0.028306053	TF, FGG, FGA, FGB, PRSS2, VEGFA, CFH, TFPI, APOH, PCSK9, SEMA3C, CP, FGL1
Extracellular matrix	2.3	2	10	6.89E-04	FLRT3, COL4A4, TF, VWF, COL4A3, MATN3, LTBP1, CTGF, PRSS2, VEGFA, VCAN, APLP1
Fibrinogen complex	2.2	4	3.3	6.98E-04	FGG, FGA, FGB, FGL1
Complement and coagulation cascades	2.2	6	5	0.01500	VWF, FGG, FGA, FGB, CFH, TFPI

^a^ Database for Annotation, Visualization and Integrated Discovery (DAVID) bioinformatics resources

^b^ number of genes involved in each functional group

^c^ percentage of genes in the functional category out of total genes in each time points

^d^ modified Fisher’s exact test (EASE score of ≤ 0.01)

In contrast to the first time point which was limited to chemokine response, DAVID analysis at 12 hpi showed that DE genes fell into diverse functional categories such as regulation of apoptosis, anti-apoptosis, wound healing and blood vessel morphogenesis (Table 4). Combination of anti-apoptotic genes such as BCL2-related protein A1 (BCL2A1) and myeloid cell leukemia sequence 1, BCL2-related (MCL1) as well as pro-apoptotic genes such as tumor necrosis factor receptor superfamily, member 21(TNFRSF21) and member 19 (TNFRSF19), and tumor necrosis factor, alpha-induced protein 3 (TNFAIP3) were induced concurrently at 6 and 12 hpi (Fig 4B). Functional analysis revealed that limited groups were enriched at 24 and 48 hpi (Table 4). Genes related to the extracellular matrix (ECM) and remodeling were affected remarkably at 24 and 48 hpi. Expression of three fibrinogen chains, α, β and γ was down regulated at 6 hpi (-1.51 β, -1.52 α), 12 hpi (-2.08 α, -2.18 β, -2.09 γ), 24 hpi (-2.22 α, -2.64 β, and -2.29 γ) and 48 hpi (-2.01 α, -2.68 β, -1.85 γ). In spite of single induction of PLAU at 24 hpi, the result demonstrated a significant increase of PLAUR at 6, 12 and 24 hpi (1.91, 1.69, 1.62 fold, respectively). The expression of other ECM related genes were induced including thrombomoduline (THBD), matrix metallopeptidase (MMP).

It is of great importance to note that in first three time points of HRV infection, the expression of dual specificity phosphatase 6 (DUSP6; type II DUSP subgroup) was up-regulated by 2.86, 2.12 and 1.50 fold at 6, 12, and 24 hpi, respectively while the expression of DUSP22 (type I DUSP subgroup) was down-regulated at 6 hpi (-1.57 fold). Analysis showed that other signal transduction related genes were found to be significantly affected including GNB4, SOCS3, SOCS2, and PREX1 (Fig 4D). Early growth response 1 (EGR1) was the most strongly up-regulated gene across all the time points (25, 5, 1.5 fold at 12, 24 and 48 hpi, respectively). In addition to EGR1, a cluster of other transcription factors including FOS-like antigen 1 (FOSL1; 3.13 fold at 6hpi; 1.88 fold at 12hpi; 1.57 fold at 24hpi), FOS-like antigen 2 (FOSL2; 1.51 fold at 6hpi), FBJ murine osteosarcoma viral oncogene homolog (FOS; 2.94 fold at 12hpi), jun proto-oncogene (JUN; 3.45 at 12hpi; 1.66 at 24hpi), v-maf musculoaponeurotic fibrosarcoma oncogene homolog F (avian) (MAFF; 2.89 at 6hpi; 1.96 at 24hpi) and ETS-domain protein (SRF accessory protein 2) (ELK3; 1.97 fold at 6 and 1.52 fold 12 hpi) were elevated also they dominated at 6, 12, and 24 hpi (Fig 4C). Collectively these data suggest that, the significant induction of chemokines preferentially at early stage could be an indication of characteristic viral infection. The prominent regulation of the apoptosis related genes together with inflammatory chemokines at early stage of infection may indicate that many important events related to the virus pathogenicity had happened during early phase of infection. Understanding the cell signaling profiles driven by DE genes can be an indication of interaction between virus and host-cell as well as prediction of cell fate.

### Selected DE genes form diverse gene ontologies showed consistent trend in RT-qPCR assay

**Fig 5:**
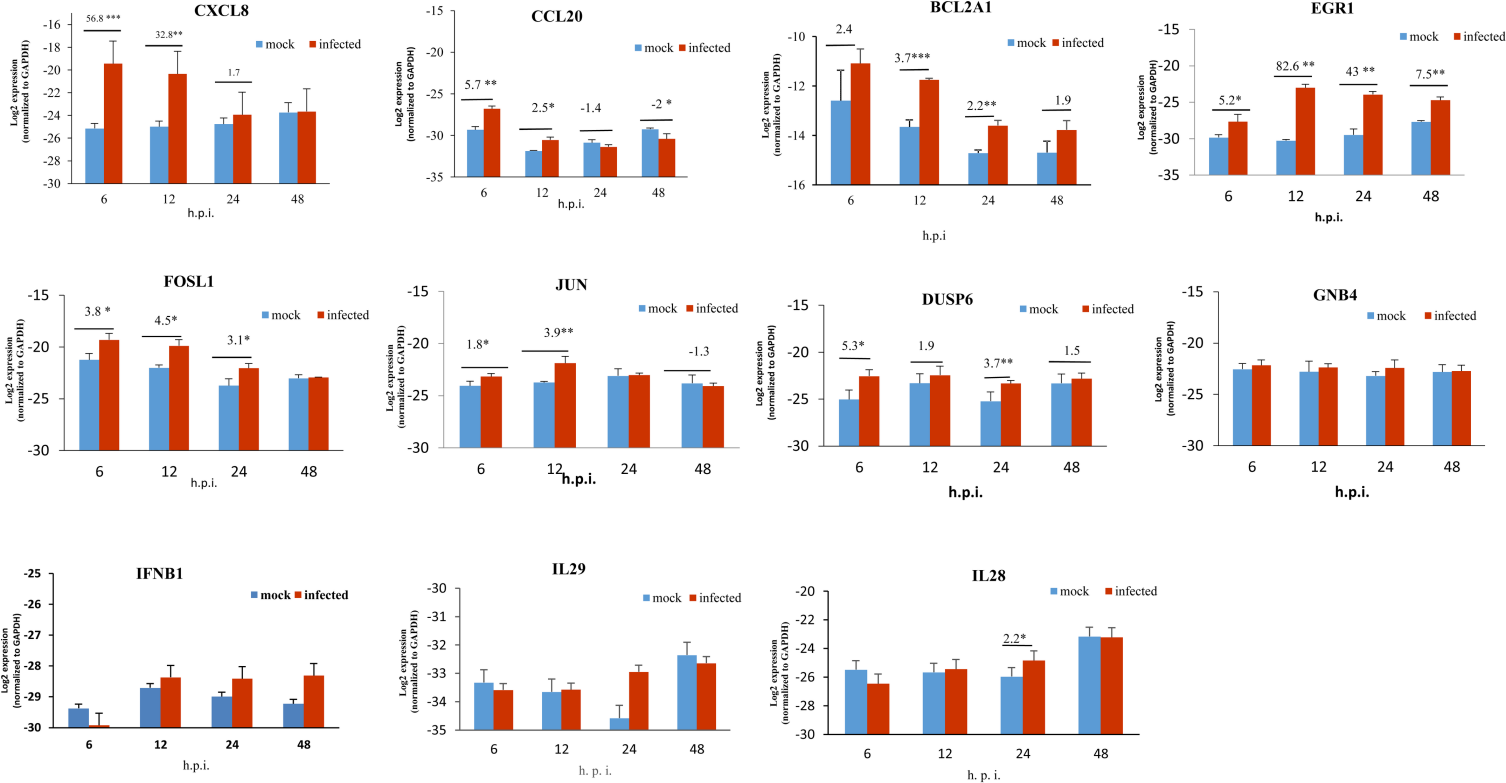
qRT-PCR validation of selected DE genes in HRV72-infected A549 cells. Eight up-regulated DEGs in microarray analysis from different functional categories and IFN genes (IFNB1, IL29 and IL28) were tested using qRT-PCR for validation. The Log_2_ values normalized to GAPDH. Fold changes are presented above the line in each time points. P< 0.05 (*) P < 0.01 (**) and P < 0.001 (***); hpi: hours post infection.

To validate the data analyzed using microarray, expression of eight DE genes from diverse functional groups including chemokine (CXCL8, CCL20, CXCL3), anti-apoptotic (BCL2A1), transcription factor (FOSL1, JUN, and EGR1) and signal transduction (DUSP6 and GNB4) was tested by RT-qPCR using same RNA samples used for microarray hybridization at 6, 12, 24 and 48 hpi. When both RT-qPCR and microarray data showed a similar direction in fold change, the genes were considered validated. Fig 5 displays the detailed analysis of 8 DE genes by RT-qPCR. In consistent with microarray, EGR1 gene showed highest fold induction based on RT-qPCR analysis (69.8 fold at 12 hpi). The results indicated that the induction of CXCL8, CCl20, CXCL3, BCL2A1, FOSL1, JUN, EGR1 and DUSP6 showed consistent fold change direction both in RT-qPCR and microarray analysis except for GNB4. However, we did not find any significant differences in the level of expression of IFN-β and IFN-λ in both microarray as well as RT-qPCR analyses.

## Discussion

The current study provides comprehensive gene expression analysis of HRV infection based on four time points using an *in vitro* system. We hypothesized that a discrete gene subsets could be disrupted along with development of infection. Undifferentiated epithelial cell culture model was selected in order to increase susceptibility of the cells to virus in order to reach uniform infection [27]. The advantage of this system allows analysis of data mainly among infected cells rather than uninfected cells. Although replication of the rhinovirus can be low grade with non-cytopathic effect [28], in the current study HRV72 infection was determined by progression of characteristic CPE which was accompanied by efficient intracellular (cell lysate) and extracellular (cell culture supernatant) accumulation of the viral genome. This finding further provides an *in vitro* evidence for implication of HRV-B in LRTI [11,28]. The transcriptional response to HRV infection was characterized by significant up- and down-regulation of the genes. The prominent down-regulation of the genes and low induction of antiviral genes could be related to the cleavage of transcriptional factors observed in *Picornaviruses* [29].

We aimed to identify the genes induced at early infection stage since modifications at early time points is mainly due to direct induction through viral signaling rather than feedback action of the other cell products which may play important role on severity and outcome of disease. In contrast to previous gene expression studies [15,17], our microarray analysis showed a significant difference between HRV-infected and mock-infected cells at 6 hpi. Gene expression analysis of air-liquid differentiated primary human tracheobronchial epithelial cells infected with HRV major and minor groups showed no DE genes at early infection stage [15]. Similar result was observed by Proud *et al*. using *in vivo* system [17]. This discrepancy might be due to patchy pattern of HRV infection *in vivo* and low susceptibility of differentiated epithelial cells for HRV infection.

The transcriptional response to HRV infection was further characterized by up-regulation of chemotactic factors at early stage. The monophasic nature of the chemokine induction started at 6 hpi and continued up to 12 hpi, potentially rejects the presence of secondary extracellular stimulus [24]. The potential role of respiratory viruses for induction of airway inflammation has been demonstrated for HRV [16] as well as other viruses [30]. The induction of chemotactic factors in this study suggests a potential role of the HRV72 infected alveolar epithelial cells to initiate pulmonary inflammation, the possible role that has been demonstrated for primary human tracheobronchial epithelial cells infected with HRV-A strains [15,17]. In addition, strain-specific differences has been reported among influenza viruses regarding the infectivity grade and inflammatory response [31]. The current study showed that HRV72 induced expression of CXC (ELR-CXC) and CC chemokines and further suggests that neutrophilic and non-neutrophilic arms of the immune response could be activated upon HRV72 infection. Indeed uncontrolled expression of the chemokines can eventually lead to pathological inflammatory reactions.

The association between CXCL8 and reduced lung function, severity of asthma and airway hyper-responsiveness [32] and allergic inflammation [33] has been demonstrated in previous studies. Our findings further support the notion that HRV-induced release of CXCL8 [32,34] and other members of CXC chemokine family (CXCL 1, 3 and 5) [35] from alveolar epithelial cells could function as neutrophil chemoatracting signal and that perpetuate the pathogenesis of the HRV-B in LRTIs. CC chemokines including CCL2 and CCL20 were significantly induced upon HRV72 infection suggesting that non-neutrophilic immune response could be potentially provoked by this virus. Association of CCL2 level with respiratory symptoms has been shown in allergic individuals [36]. Previous studies demonstrated that CCL2 was implicated in HRV induced airway hyper-responsiveness as a mechanism underlying asthma exacerbation [36,37]. Further *in vivo* studies would address the relationship between immune parameters e.g. chemokine level and severity of the disease.

Contrary to expectations that induction of type I IFN and subsequent expression of interferon-stimulated genes (ISGs) are common phenomenon in HRV infected primary human tracheobronchial epithelial cells [15,17], this study did not find a significant induction of IFN mRNA expression. The induction of IFN-β mRNA and activation of Jak/STAT pathway was not also reported for HRV 14 [20,38]. It has been shown that IRF3 acts as an intermediate molecule to stimulate the induction of IFN-I and variety of ISGs in airway epithelial cells infected with HRV group A [39]. Low level of the IFN-I in the study by Kotla et l. (2008) [38] could be explained by the impaired activation of the IRF3 in the presence of the activated NF-κB and ATF-2. Variable grades of IFN type I induction in the strain-specific differences manner have been shown among influenza viruses and host antiviral which could play a critical role in clinical disease [31,40]. The discrepancy in the induction or inhibition of IFN-I observed in HRV infections is not clear and could be different between primary and immortalized cells as well as different virus serotypes. This finding further emphasizes the important implication of developing IFN based treatment for HRV-B infections. Defective induction of major antiviral genes could be primarily related to the defective induction of IFN genes. HRV72 induced expression of tripartite motif containing (TRIM 10, 68, 26, and 69) genes, which play a broad role in innate immune response. The positive regulatory effect versus negative regulatory effect of TRIM proteins on antiviral response has been reported in other viruses and it is noteworthy to investigate the effect of TRIMs found in this study on HRV infection [41].

Viruses have evolved strategies to exploit cell signaling pathways for virus replication. Targeting of these pathways might be a valuable antiviral approach [42]. MAPK signaling pathways play critical role in innate and adaptive immune response. Association of MAPK activation to the production of chemokines and cytokines has been shown in influenza infection[42]. Inactivation of MAPK pathways through DUSP also plays an important role in immune system[43]. Interestingly, DUSP6 was induced at first three time points in our study. Understanding the role of DUSP6 in MAPKs regulation in the context of HRV infection may require further investigations. Activation of signal transduction pathways could result in activation of transcription factors such as AP-1 and NF-κB. HRV72 induced expression of FOSL1, FOSL2, FOS and JUN genes. Combinations of the Fos and Jun proteins constitute dimeric transcription factor called AP-1. The AP-1 mediated production of chemokies has been demonstrated by Hoffmann *et al*. [44] who delineated different roles for Fra-1 and c-Fos in transcription of IL8. Cooperative role of AP-1 in conjugation with NF-κB in IL8 induction has been shown for RSV[45]. More research on this topic including protein level expression as well as determination of absolute or cooperative interaction of AP-1 with other transcription factors in stimulation of chemokines could be useful for the develop of combinatorial therapy for HRV infection. Surprisingly EGR-1 was the DE gene expressed at the highest level in this study. Previous study reveal that HSV-1 viral load and subsequent mortality is reduced in EGR-1 knockout mice suggestive of EGR-1 role in regulation of virus replication [46]. Further investigations would address the association between EGR-1 and HRV72 replication.

Genes involved in regulation of pro- versus anti-apoptotic responses were differentially expressed upon HRV infection in airway epithelial cells. The previous studies of apoptotic pathways indicated that different HRV strains can activate both intrinsic and extrinsic apoptotic pathways through caspase 9 [47] and caspase 8, respectively [48]. BCL2A and MCL1 belonged to the anti-apoptotic regulators of Bcl-2 family [49] were significantly induced in response to HRV infection. The cytoprotective effect of over-expressed BCL2A and MCL1 genes played critical role in neutrophil survival [50] and inhibited oxidant-induced necrosis and apoptosis in lung epithelial cells[51]. The induction of BCL2A1 and MCL-1 may be causing higher cell viability despite incomplete preservation of cell morphology and development of CPE in the current study. Over-expressed Bcl-2 and Bcl-xl in Coxsackievirus B3 infected cells preserved high cell viability and inhibited release of Cyt c from mitochondria [52]. From a translational point of view, in depth understanding of pro/anti-apoptotic pathways regulated by the virus could lead to the development of novel therapeutics against HRV.

The category of the genes related to the wound healing and coagulation cascade were differentially expressed between HRV infected vs. un-infected airway epithelia cells. The expression of fibrinogen chains, α, β and γ, was down-regulated during the infection course, although the finding was different from previous studies using primary human epithelial cells [15,17]. The over expression of fibrinogen was demonstrated in infected lung epithelial cells which could contribute to pathology of lung damage [53,54]. PLAU/PLAUR system play complex role in airway system such as cellular adhesion, proliferation, migration and tissue remodeling [55]. PLAU/PLAUR system was induced in current study, the finding that is inconsistent with previous reports [15,17]. PLAUR plays a role in infiltration of immune cell is lung bacterial infection although didn’t show similar role in RSV and influenza using murine models [56]. Coagulation plays an essential role in control and clearance of the infection. The inta-alveolar deposition of fibrin by epithelial cells is a typical response to the lung injury and infection[57]. On the other hand, the expression of the coagulation cascade related genes including THBD and MMP were induced here.

The study showed detail analysis of HRV-B/cell interaction using A549 cells derived from a human alveolar carcinoma cells with properties of type II alveolar epithelial cells. The finding based on the model, however, must be carefully interpreted and cautiously extrapolated to the real airway system in human. It is not clear to what extent HRV-B induced inflammatory responses are involved in protective or immunopathogenic features of the infection. The relative role of the immune factors in clearing versus pathology of the virus may also have like to patient underlying condition. The chemokine role in viral infection can be further characterized by analysing chemokine knockout mice in the context of infection. It is intriguing to speculate that defective elimination of HRV-B72 in the current in vitro system might be presented as a severe case in *in vivo* system. Further research regarding to the polymorphism of chemokine genes would be advantageous in determining genotype-phenotype associations of HRV infections and complications [58].

In summary, comprehensive gene expression analysis of human lung epithelial cells infected with HRV-B was presented in current study. Various functional gene categories were enriched at different time points post infection, which might be specific for HRVB species. This study provides a foundation for the further investigation of the genes and pathways that might be involved in the pathogenesis of the HRVB and the possible targets for treatment.

## Supporting information

S1 Fig SDS page analysis of purified HRV72 virus. 20 μl of each sucrose fractions were mixed with 20 μl of 1× SDS page sample buffer and denatured for 10 min at 100°C followed by gel running and subsequent staining of SDS page with coomassie blue. Sucrose fractions from up to down were run onto the gel from right to left order. In the UP6 fraction, the purified virion shows three distinct bands which is more likely corresponded to vp1-3.
(TIF)

S2 Fig HRV72 plaque formation on H1Hela cells. H1Hela cells were seeded in 6-well plates at a cell density of 4–5 × 10^5^ cells per well and incubated overnight at 37°C. Wells infected with 0.1 ml of dilutions of virus ranging from 10^1^ (left up) to 10^−10^ and a mock-infected (right down) in duplicates. At the end of 5-day incubation period at 34°C, the carboxymethylcellulose over layer was decanted and stained with 0.1% crystal violet solution. Plaques were counted in the 10^−7^ and 10^−8^ wells using this equation: PFU/ml = (Mean of plaques / amount of inoculum (ml)) / well dilution.
(TIF)

S3 FigToxicity of HRV72 on A549 cells.Cells were seeded at optimal density of 1×10^4^ cells/well in triplicates in 96 well plate and the cell infection was carried out at 24 h when cells were almost 80% confluent. The cells were inoculated with various MOI (1, 5, 7, 10, 15, 20, 40 and 100 pfu/mL) and incubated at 34°C with 5% CO_2_. Toxicity was measured by determining metabolic activity of the A549 cells using MTT assay. Data are presented as average of three independent experiments. The cell viability was calculated with this equation: $=\frac{absorbance\;of\;sample}{absorbance\;of\;negative\;control}\times 100$. The absorbance was measured at 570 nm.(TIF)

S1 Table The complete list of DE genes with ≥1.5 fold-change at 6 hpi. DE genes were generated by Transcriptome Analysis Console v2.0. p-values obtained from One-Way Between-Subject ANOVA (unpaired).
(XLSX)

S2 Table The complete list of DE genes with ≥1.5 fold-change at 12 hpi. DE genes were generated by Transcriptome Analysis Console v2.0. p-values obtained from One-Way Between-Subject ANOVA (unpaired).
(XLSX)

S3 Table The complete list of DE genes with ≥1.5 fold-change at 24 hpi. DE genes were generated by Transcriptome Analysis Console v2.0. p-values obtained from One-Way Between-Subject ANOVA (unpaired).
(XLSX)

S4 Table The complete list of DE genes with ≥1.5 fold-change at 48 hpi. DE genes were generated by Transcriptome Analysis Console v2.0. p-values obtained from One-Way Between-Subject ANOVA (unpaired).
(XLSX)

S5 Table The complete list of overlap genes with ≥1.5 fold-change. The overlaps of DE genes have been revealed across all-time points using Venn diagrams.
(XLSX)

## References

[1] Racaniello VR (2013) Picornaviridae: The Viruses and Their Replication In: Knipe DM, Howley PM, editors. FIELDS VIROLOGY. Sixth ed. Philadelphia, PA 19103 USA: Wolters Kluwer Lippincott Williams & Wilkins pp. 453–489.

[2] Kusel MM, de Klerk NH, Holt PG, Kebadze T, Johnston SL, Sly PD (2006) Role of respiratory viruses in acute upper and lower respiratory tract illness in the first year of life: a birth cohort study. The Pediatric infectious disease journal 25: 680–686. doi: 10.1097/01.inf.0000226912.88900.a3 16874165 10.1097/01.inf.0000226912.88900.a3

[3] McIntyre CL, Knowles NJ, Simmonds P (2013) Proposals for the classification of human rhinovirus species A, B and C into genotypically assigned types. Journal of General Virology 94: 1791–1806. doi: 10.1099/vir.0.053686-0 23677786 10.1099/vir.0.053686-0 PMC3749525

[4] Pitkäranta A, Arruda E, Malmberg H, Hayden FG (1997) Detection of rhinovirus in sinus brushings of patients with acute community-acquired sinusitis by reverse transcription-PCR. Journal of clinical microbiology 35: 1791–1793. 9196195 10.1128/jcm.35.7.1791-1793.1997 PMC229843

[5] Blomqvist S, Roivainen M, Puhakka T, Kleemola M, Hovi T (2002) Virological and serological analysis of rhinovirus infections during the first two years of life in a cohort of children. Journal of medical virology 66: 263–268. 11782938 10.1002/jmv.2140

[6] Aponte FE, Taboada B, Espinoza MA, Arias-Ortiz MA, Monge-Martínez J, Rodríguez-Vázquez R, et al (2015) Rhinovirus is an important pathogen in upper and lower respiratory tract infections in Mexican children. Virology journal 12: 1. 25889995 10.1186/s12985-015-0262-z PMC4349319

[7] Papadopoulos NG (2004) Do rhinoviruses cause pneumonia in children? Paediatric respiratory reviews 5: S191–S195. 14980269 10.1016/s1526-0542(04)90036-x

[8] Peltola V, Jartti T, Putto‐Laurila A, Mertsola J, Vainionpää R, Waris M, et al (2009) Rhinovirus infections in children: A retrospective and prospective hospital‐based study. Journal of medical virology 81: 1831–1838. doi: 10.1002/jmv.21590 19697407 10.1002/jmv.21590 PMC7166645

[9] Halperin SA, Eggleston PA, Beasley P, Suratt P, Hendley JO, Gröschel DH, et al (1985) Exacerbations of Asthma in Adults during Experimental Rhinovirus Infection 1–3. American Review of Respiratory Disease 132: 976–980. doi: 10.1164/arrd.1985.132.5.976 2998246 10.1164/arrd.1985.132.5.976

[10] Gern JE, Galagan DM, Jarjour NN, Dick EC, Busse WW (1997) Detection of rhinovirus RNA in lower airway cells during experimentally induced infection. American journal of respiratory and critical care medicine 155: 1159–1161. doi: 10.1164/ajrccm.155.3.9117003 9117003 10.1164/ajrccm.155.3.9117003

[11] Imakita M, Shiraki K, Yutani C, Ishibashi-Ueda H (2000) Pneumonia Caused by Rhinovirus. Clinical Infectious Diseases 30: 611–612. doi: 10.1086/313723 10722460 10.1086/313723

[12] Mosser AG, Brockman-Schneider R, Amineva S, Burchell L, Sedgwick JB, Busse WW, et al (2002) Similar Frequency of Rhinovirus-Infectible Cells in Upper and Lower Airway Epithelium. Journal of Infectious Diseases 185: 734–743. doi: 10.1086/339339 11920291 10.1086/339339

[13] Wark P, Johnston S, Moric I, Simpson J, Hensley M, Gibson P (2001) Neutrophil degranulation and cell lysis is associated with clinical severity in virus-induced asthma. European Respiratory Journal 19: 68–75. 10.1183/09031936.02.00226302 11852895

[14] Proud D (2011) Role of rhinovirus infections in asthma. Asian Pacific Journal of Allergy and Immunology 29: 201–208. 22053589

[15] Chen Y, Hamati E, Lee PK, Lee WM, Wachi S, Schnurr D, et al (2006) Rhinovirus induces airway epithelial gene expression through double-stranded RNA and IFN-dependent pathways. American journal of respiratory cell and molecular biology 34: 192 doi: 10.1165/rcmb.2004-0417OC 16210696 10.1165/rcmb.2004-0417OC PMC2644182

[16] Bochkov YA, Hanson KM, Keles S, Brockman-Schneider RA, Jarjour NN, Gern JE (2009) Rhinovirus-induced modulation of gene expression in bronchial epithelial cells from subjects with asthma. Mucosal Immunol 3: 69–80. doi: 10.1038/mi.2009.109 19710636 10.1038/mi.2009.109 PMC2884103

[17] Proud D, Turner RB, Winther B, Wiehler S, Tiesman JP, Reichling TD, et al (2008) Gene Expression Profiles during In Vivo Human Rhinovirus Infection. American Journal of Respiratory and Critical Care Medicine 178: 962–968. doi: 10.1164/rccm.200805-670OC 18658112 10.1164/rccm.200805-670OC

[18] Lau SK, Yip CC, Lin AW, Lee RA, So L-Y, Lau Y-L, et al (2009) Clinical and molecular epidemiology of human rhinovirus C in children and adults in Hong Kong reveals a possible distinct human rhinovirus C subgroup. Journal of Infectious Diseases 200: 1096–1103. doi: 10.1086/605697 19708791 10.1086/605697 PMC7199882

[19] Etemadi MR, Othman N, Savolainen-Kopra C, Sekawi Z, Wahab N, Sann LM (2013) Biodiversity and clinico-demographic characteristics of human rhinoviruses from hospitalized children with acute lower respiratory tract infections in Malaysia. Journal of Clinical Virology 58: 671–677. doi: 10.1016/j.jcv.2013.05.017 23932333 10.1016/j.jcv.2013.05.017 PMC7172529

[20] Peng T, Kotla S, Bumgarner RE, Gustin KE (2006) Human Rhinovirus Attenuates the Type I Interferon Response by Disrupting Activation of Interferon Regulatory Factor 3. Journal of Virology 80: 5021–5031. doi: 10.1128/JVI.80.10.5021-5031.2006 16641293 10.1128/JVI.80.10.5021-5031.2006 PMC1472094

[21] Schuler BA, Schreiber MT, Li L, Mokry M, Kingdon ML, Raugi DN, et al (2014) Major and minor group rhinoviruses elicit differential signaling and cytokine responses as a function of receptor-mediated signal transduction. PloS one 9: e93897 doi: 10.1371/journal.pone.0093897 24736642 10.1371/journal.pone.0093897 PMC3988043

[22] Dolan TM, Fenters JD, Fordyce PA, Holper JC (1968) Rhinovirus plaque formation in WI-38 cells with methylcellulose overlay. Applied microbiology 16: 1331–1336. 4300170 10.1128/am.16.9.1331-1336.1968 PMC547650

[23] Walum E, Stenberg K, Jenssen D (1991) Understanding cell toxicology: principles and practice Ellis Horwood. J Appl Toxicol 11: 389–390.

[24] Griego SD, Weston CB, Adams JL, Tal-Singer R, Dillon SB (2000) Role of p38 Mitogen-Activated Protein Kinase in Rhinovirus-Induced Cytokine Production by Bronchial Epithelial Cells. The Journal of Immunology 165: 5211–5220. 11046054 10.4049/jimmunol.165.9.5211

[25] Irizarry RA, Bolstad BM, Collin F, Cope LM, Hobbs B, Speed TP (2003) Summaries of Affymetrix GeneChip probe level data. Nucleic acids research 31: e15–e15. 12582260 10.1093/nar/gng015 PMC150247

[26] AFFYMETRIX (2003). Genechip Expression Analysis Technical Manual. http://www.affymetrix.com/support/,technical/manual/expression manual.affx. Santa Clara, CA: Affymetrix.

[27] Lopez-Souza N, Dolganov G, Dubin R, Sachs LA, Sassina L, Sporer H, et al (2004) Resistance of differentiated human airway epithelium to infection by rhinovirus. American Journal of Physiology-Lung Cellular and Molecular Physiology 286: L373–L381. doi: 10.1152/ajplung.00300.2003 14711802 10.1152/ajplung.00300.2003

[28] Johnston SL, Papi A, Bates PJ, Mastronarde JG, Monick MM, Hunninghake GW (1998) Low Grade Rhinovirus Infection Induces a Prolonged Release of IL-8 in Pulmonary Epithelium. The Journal of Immunology 160: 6172–6181. 9637536

[29] Lyles DS (2000) Cytopathogenesis and inhibition of host gene expression by RNA viruses. Microbiology and molecular biology reviews 64: 709–724. 11104816 10.1128/mmbr.64.4.709-724.2000 PMC99011

[30] Zhang Y, Luxon BA, Casola A, Garofalo RP, Jamaluddin M, Brasier AR (2001) Expression of respiratory syncytial virus-induced chemokine gene networks in lower airway epithelial cells revealed by cDNA microarrays. Journal of Virology 75: 9044–9058. doi: 10.1128/JVI.75.19.9044-9058.2001 11533168 10.1128/JVI.75.19.9044-9058.2001 PMC114473

[31] Patel JR, Vora KP, Tripathi S, Zeng H, Tumpey TM, Katz JM, et al (2011) Infection of lung epithelial cells with pandemic 2009 A (H1N1) influenza viruses reveals isolate-specific differences in infectivity and host cellular responses. Viral immunology 24: 89–99. doi: 10.1089/vim.2010.0122 21449719 10.1089/vim.2010.0122

[32] Grünberg K, Timmers M, Smits H, Klerk E, Dick E, Spaan W, et al (1997) Effect of experimental rhinovirus 16 colds on airway hyperresponsiveness to histamine and interleukin‐8 in nasal lavage in asthmatic subjects in vivo. Clinical & Experimental Allergy 27: 36–45. 10.1111/j.1365-2222.1997.tb00670.x PMC7164827 9117878

[33] Shute J (1994) Interleukin‐8 is a potent eosinophil chemo‐attractant. Clinical & Experimental Allergy 24: 203–206. 8012851 10.1111/j.1365-2222.1994.tb00220.x

[34] Chun YH, Park JY, Lee H, Kim HS, Won S, Joe HJ, et al (2013) Rhinovirus-infected epithelial cells produce more IL-8 and RANTES compared with other respiratory viruses. Allergy, asthma & immunology research 5: 216–223. 10.4168/aair.2013.5.4.216 PMC3695236 23814675

[35] Becker S, Quay J, Koren HS, Haskill J (1994) Constitutive and stimulated MCP-1, GRO alpha, beta, and gamma expression in human airway epithelium and bronchoalveolar macrophages. American Journal of Physiology-Lung Cellular and Molecular Physiology 266: L278–L286. 10.1152/ajplung.1994.266.3.L278 8166297

[36] Lewis TC, Henderson TA, Carpenter AR, Ramirez IA, McHenry CL, Goldsmith AM, et al (2012) Nasal cytokine responses to natural colds in asthmatic children. Clinical & Experimental Allergy 42: 1734–1744. 23181789 10.1111/cea.12005 PMC4219353

[37] Schneider D, Hong JY, Bowman ER, Chung Y, Nagarkar DR, McHenry CL, et al (2013) Macrophage/epithelial cell CCL2 contributes to rhinovirus-induced hyperresponsiveness and inflammation in a mouse model of allergic airways disease. American Journal of Physiology-Lung Cellular and Molecular Physiology 304: L162–L169. doi: 10.1152/ajplung.00182.2012 23204071 10.1152/ajplung.00182.2012 PMC3567365

[38] Kotla S, Peng T, Bumgarner RE, Gustin KE (2008) Attenuation of the type I interferon response in cells infected with human rhinovirus. Virology 374: 399–410. doi: 10.1016/j.virol.2008.01.022 18272195 10.1016/j.virol.2008.01.022

[39] Wang Q, Nagarkar DR, Bowman ER, Schneider D, Gosangi B, Lei J, et al (2009) Role of double-stranded RNA pattern recognition receptors in rhinovirus-induced airway epithelial cell responses. The Journal of Immunology 183: 6989–6997. doi: 10.4049/jimmunol.0901386 19890046 10.4049/jimmunol.0901386 PMC2920602

[40] Hayman A, Comely S, Lackenby A, Murphy S, McCauley J, Goodbourn S, et al (2006) Variation in the ability of human influenza A viruses to induce and inhibit the IFN-β pathway. Virology 347: 52–64. doi: 10.1016/j.virol.2005.11.024 16378631 10.1016/j.virol.2005.11.024

[41] Kawai T, Akira S (2011) Toll-like receptors and their crosstalk with other innate receptors in infection and immunity. Immunity 34: 637–650. doi: 10.1016/j.immuni.2011.05.006 21616434 10.1016/j.immuni.2011.05.006

[42] Ludwig S (2007) Influenza viruses and MAP kinase cascades–Novel targets for an antiviral intervention? Signal Transduction 7: 81–88.

[43] Lang R, Hammer M, Mages J (2006) DUSP meet immunology: dual specificity MAPK phosphatases in control of the inflammatory response. The Journal of Immunology 177: 7497–7504. 17114416 10.4049/jimmunol.177.11.7497

[44] Hoffmann E, Thiefes A, Buhrow D, Dittrich-Breiholz O, Schneider H, Resch K, et al (2005) MEK1-dependent Delayed Expression of Fos-related Antigen-1 Counteracts c-Fos and p65 NF-κB-mediated Interleukin-8 Transcription in Response to Cytokines or Growth Factors. Journal of Biological Chemistry 280: 9706–9718. doi: 10.1074/jbc.M407071200 15615716 10.1074/jbc.M407071200

[45] Mastronarde JG, Monick MM, Mukaida N, Matsushima K, Hunninghake GW (1998) Activator protein-1 is the preferred transcription factor for cooperative interaction with nuclear factor-κB in respiratory syncytial virus-induced interleukin-8 gene expression in airway epithelium. Journal of Infectious Diseases 177: 1275–1281. 9593012 10.1086/515279

[46] Chen S-H, Yao H-W, Chen I-T, Shieh B, Li C, Chen S-H (2008) Suppression of transcription factor early growth response 1 reduces herpes simplex virus lethality in mice. The Journal of clinical investigation 118: 3470 doi: 10.1172/JCI35114 18769632 10.1172/JCI35114 PMC2525697

[47] Deszcz L, Gaudernak E, Kuechler E, Seipelt J (2005) Apoptotic events induced by human rhinovirus infection. Journal of general virology 86: 1379–1389. doi: 10.1099/vir.0.80754-0 15831950 10.1099/vir.0.80754-0

[48] Taimen P, Berghäll H, Vainionpää R, Kallajoki M (2004) NuMA and nuclear lamins are cleaved during viral infection—inhibition of caspase activity prevents cleavage and rescues HeLa cells from measles virus-induced but not from rhinovirus 1B-induced cell death. Virology 320: 85–98. doi: 10.1016/j.virol.2003.11.026 15003865 10.1016/j.virol.2003.11.026

[49] Elmore S (2007) Apoptosis: a review of programmed cell death. Toxicologic pathology 35: 495–516. doi: 10.1080/01926230701320337 17562483 10.1080/01926230701320337 PMC2117903

[50] Moulding DA, Akgul C, Derouet M, White MR, Edwards SW (2001) BCL-2 family expression in human neutrophils during delayed and accelerated apoptosis. Journal of leukocyte biology 70: 783–792. 11698499

[51] He CH, Waxman AB, Lee CG, Link H, Rabach ME, Ma B, et al (2005) Bcl-2–related protein A1 is an endogenous and cytokine-stimulated mediator of cytoprotection in hyperoxic acute lung injury. Journal of Clinical Investigation 115: 1039 doi: 10.1172/JCI23004 15841185 10.1172/JCI23004 PMC1070412

[52] Carthy CM, Yanagawa B, Luo H, Granville DJ, Yang D, Cheung P, et al (2003) Bcl-2 and Bcl-xL overexpression inhibits cytochrome c release, activation of multiple caspases, and virus release following coxsackievirus B3 infection. Virology 313: 147–157. 12951029 10.1016/s0042-6822(03)00242-3

[53] Tan Y-J, Tham P-Y, Chan DZ, Chou C-F, Shen S, Fielding BC, et al (2005) The severe acute respiratory syndrome coronavirus 3a protein up-regulates expression of fibrinogen in lung epithelial cells. Journal of virology 79: 10083–10087. doi: 10.1128/JVI.79.15.10083-10087.2005 16014971 10.1128/JVI.79.15.10083-10087.2005 PMC1181587

[54] Haidaris P, Hui Z, Wright T, Neroni L, Earnest B, Courtney M (1992) Extrahepatic expression of the γ chain of fibrinogen during an acute phase response. Blood 80: 307a.

[55] Blasi F, Carmeliet P (2002) uPAR: a versatile signalling orchestrator. Nature reviews Molecular cell biology 3: 932–943. doi: 10.1038/nrm977 12461559 10.1038/nrm977

[56] Ramos M, Lao Y, Eguiluz C, Del Val M, Martínez I (2015) Urokinase receptor-deficient mice mount an innate immune response to and clarify respiratory viruses as efficiently as wild-type mice. Virulence 6: 710–715. doi: 10.1080/21505594.2015.1057389 26115163 10.1080/21505594.2015.1057389 PMC4720239

[57] Wang L, Bastarache JA, Wickersham N, Fang X, Matthay MA, Ware LB (2007) Novel role of the human alveolar epithelium in regulating intra-alveolar coagulation. American journal of respiratory cell and molecular biology 36: 497–503. doi: 10.1165/rcmb.2005-0425OC 17099142 10.1165/rcmb.2005-0425OC PMC1899324

[58] Doyle WJ, Casselbrant ML, Li-Korotky H-S, Doyle APC, Lo C-Y, Turner R, et al (2010) The interleukin 6− 174 C/C genotype predicts greater rhinovirus illness. Journal of Infectious Diseases 201: 199–206. doi: 10.1086/649559 20001857 10.1086/649559 PMC2943745

