# Supplementary figures and images for: Gene expression patterns induced at different stages of rhinovirus infection in human alveolar epithelial cells
Source: PLoS One. 2017 May 30;12(5):e0176947. doi: 10.1371/journal.pone.0176947 (PMC5448745; doi:10.1371/journal.pone.0176947)

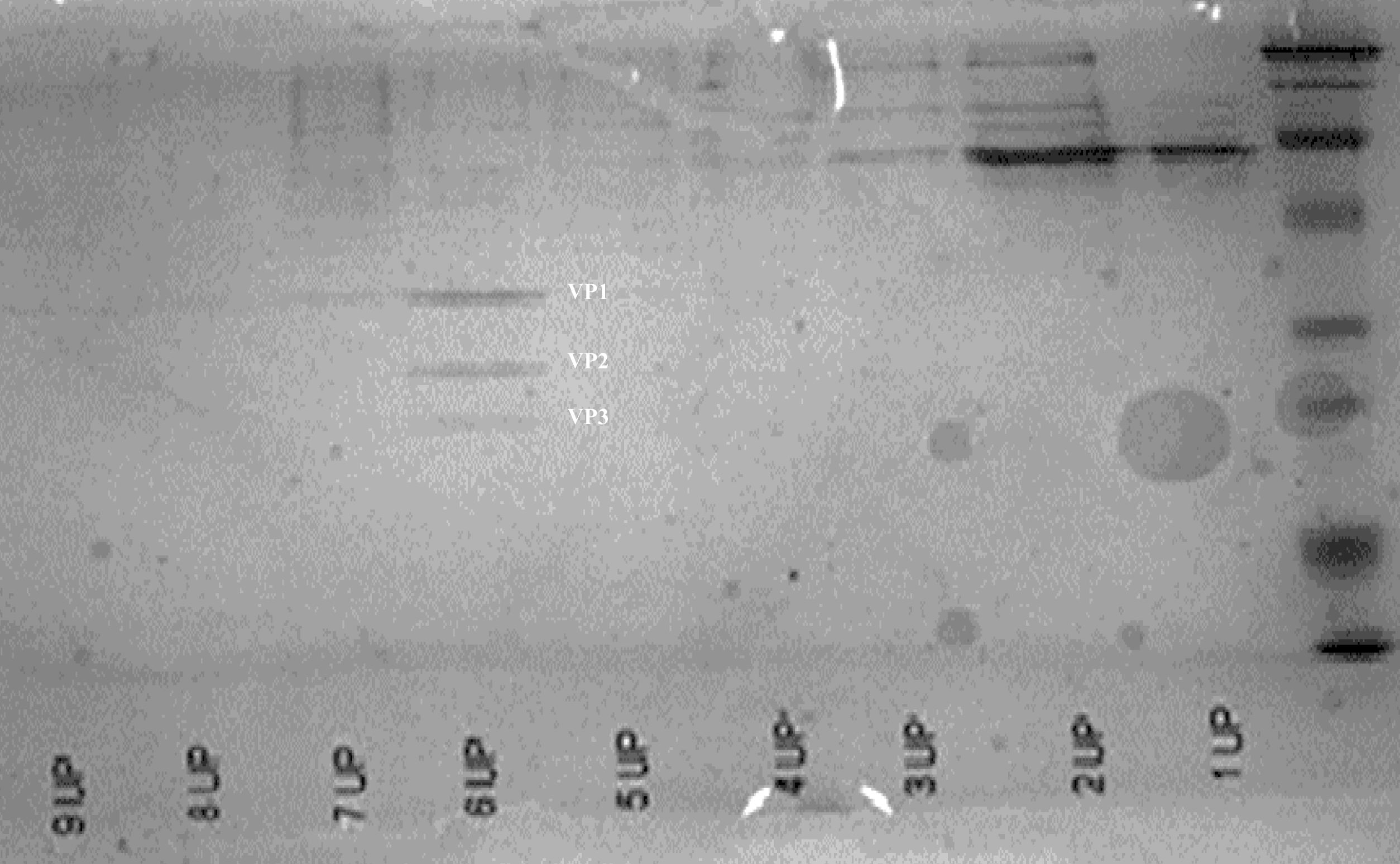

Supplement: S1 Fig — 20 μl of each sucrose fractions were mixed with 20 μl of 1× SDS page sample buffer and denatured for 10 min at 100°C followed by gel running and subsequent staining of SDS page with coomassie blue. Sucrose fractions from up to down were run onto the gel from right to left order. In the UP6 fraction, the purified virion shows three distinct bands which is more likely corresponded to vp1-3. (TIF) [file pone.0176947.s001.tif]

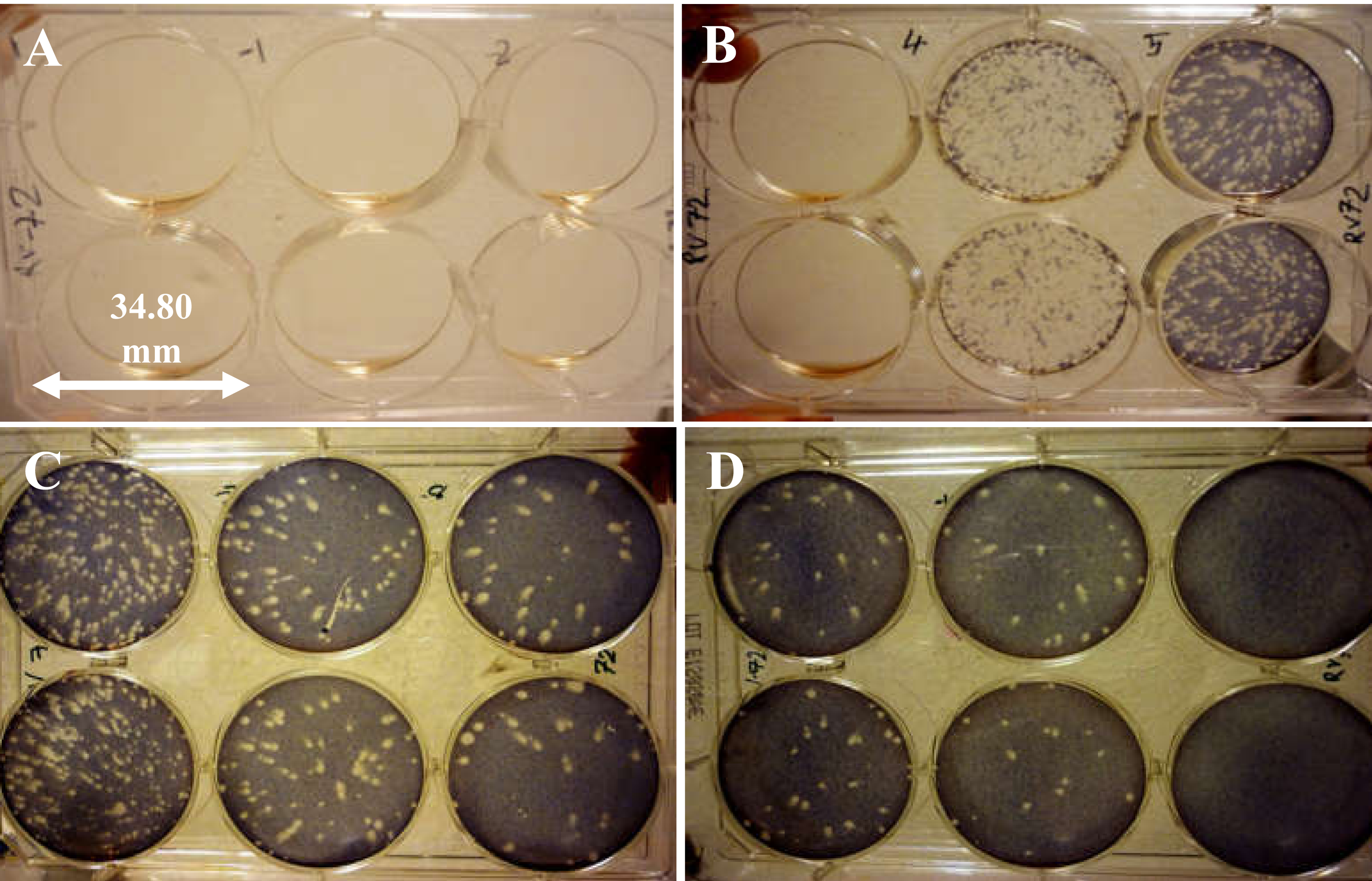

Supplement: S2 Fig — H1Hela cells were seeded in 6-well plates at a cell density of 4–5 × 10^5^ cells per well and incubated overnight at 37°C. Wells infected with 0.1 ml of dilutions of virus ranging from 101 (left up) to 10−10 and a mock-infected (right down) in duplicates. At the end of 5-day incubation period at 34°C, the carboxymethylcellulose over layer was decanted and stained with 0.1% crystal violet solution. Plaques were counted in the 10−7 and 10−8 wells using this equation: PFU/ml = (Mean of plaques / amount of inoculum (ml)) / well dilution. (TIF) [file pone.0176947.s002.tif]

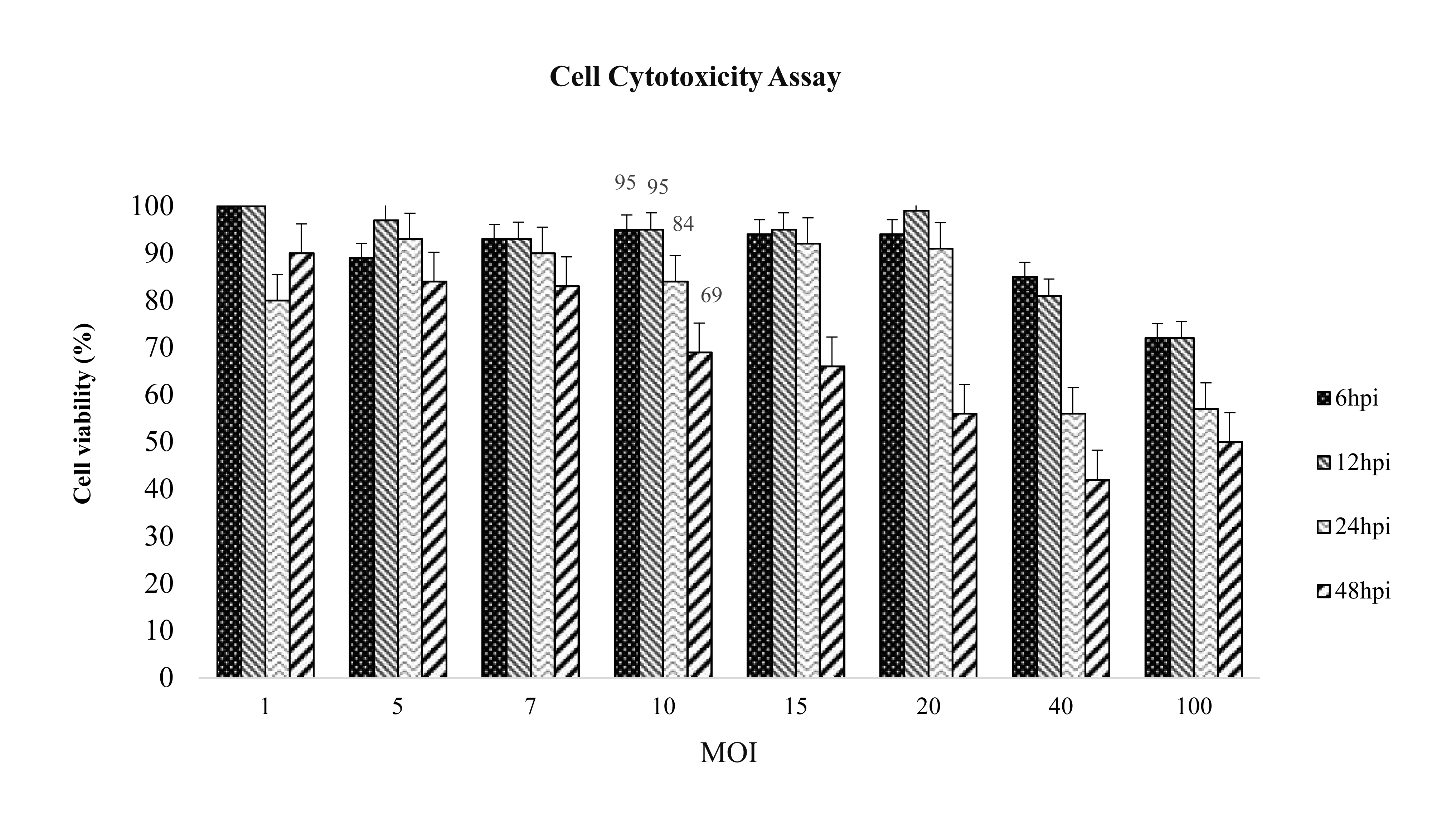

Supplement: S3 Fig — Cells were seeded at optimal density of 1×10^4^ cells/well in triplicates in 96 well plate and the cell infection was carried out at 24 h when cells were almost 80% confluent. The cells were inoculated with various MOI (1, 5, 7, 10, 15, 20, 40 and 100 pfu/mL) and incubated at 34°C with 5% CO2. Toxicity was measured by determining metabolic activity of the A549 cells using MTT assay. Data are presented as average of three independent experiments. The cell viability was calculated with this equation: =absorbanceofsampleabsorbanceofnegativecontrol×100. The absorbance was measured at 570 nm. (TIF) [file pone.0176947.s003.tif]
